# The London Poor

**Published:** 1891-01-31

**Authors:** 


### The London Poor.

London has provided excellent wards for wayfarers, and these offer 1,800 beds nightly, each place clean, warm, and retired, in which the unfortunate wayfarer, man, woman, or child, is able to claim admittance, and share the charity of London without association. The relief is good, and both supper and other meals are provided, as well as the means of cleanliness —in fact, cleanliness is enforced. During all the severe weather of late, these wards have not nearly been filled, indeed, more than half of the whole have been empty, for the want of occupants. Within a few minutes' walk of any of the Thames bridges any houseless person could have obtained, without any waiting in the cold, or any question, admittance to a ward, with a bath, change of clothes, supper, private bedroom, and breakfast in the morning, the sole return exacted being such a task of labour as it would be within his ability to give. The number of admittances to these places have been about 800 each night, leaving about a thousand vacancies. The last return shows that there were 688 men, 117 women, and four children as the inmates of these wards one night lately, as compared with 853 on the corresponding night last year, when there were 705 men, 134 women, and 14 children inmates. In regard to London pauperism generally, the advance of thrift among the population is shown by the fact that, notwithstanding the exceptional period of two months of hard weather, which has thrown vast numbers of industrious persons out of work, there were, at the date of the last return, only 109 more persons on the relief rates in London than last year, when the weather was open and trade was flourishing. In most parts of London there is actually a decrease as compared with last year. For instance, in the eastern parishes the number of all classes of pauper poor in Shoreditch, Bethnal Green, Whitechapel, St. George's-in-the-East, Stepney, Mile End Old Town, and Poplar, numbered 16,852, as compared with 17,410 last year. "East end distress," therefore, cannot be seen from the Poor Law returns. In the western districts, comprising the parishes from the Houses of Parliament to Fulham, there is also a decrease, the numbers in the middle of this month being 12,181, as compared with 12,667 at the same period last year. In the central districts comprising St. Giles's and St. George's (Bloomsbury), the Strand Union, the Holborn Union, and the City of London, the total pauperism at the same period was 12,146, while last year, at the corresponding period, it stood at 12,953. The South London district shows the worst feature of London pauperism, not because the poverty is more acute there, but because the administration, in most cases, is careless.

